# Champions as catalysts for change: a realist evaluation of their impact in primary care

**DOI:** 10.1186/s12913-025-13573-0

**Published:** 2025-11-18

**Authors:** Elisabeth Martin, Dave Bergeron, Isabelle Gaboury

**Affiliations:** 1 Département de médecine de famille, Université de Sherbrooke, 150, place Charles-Le Moyne, C. P. 200, Longueuil, QC J4K 0A8 Canada; 2 Département des sciences de la santé, Université du Québec à Rimouski, Rimouski, Canada

**Keywords:** Champion, Quality improvement, Primary Healthcare clinic, Realist Evaluation

## Abstract

**Background:**

The healthcare system, particularly primary care, is constantly evolving to adapt to technological innovations and demographic changes. Quality improvement (QI) initiatives are a key strategy for optimizing care processes and better meeting patient needs. However, their success depends on several contextual factors, including alignment with organizational priorities, resource availability, and stakeholder engagement. While champions are widely recognized as important for change within organizations, the specific causal relationship of their impact remain unclear. This study aims to deepen understanding of champions’ behaviours in the QI process within interdisciplinary primary care settings.

**Method:**

A multiple case study was conducted using a realist evaluation approach to refine and validate the program theory. Data collection involved document analysis and individual semi-structured interviews. Context-Mechanism-Outcome (CMO) configurations were constructed to explore relationships between context (pre-existing elements shaping interventions), mechanisms (processes activated in a given context), and outcomes to iteratively refine the program theory. The study was conducted in primary healthcare clinics, the predominant model of primary care clinics in Quebec, Canada. Data were collected between 2022 and 2023 from four cases of champions in Quebec and Ontario who had implemented a change using a QI approach.

**Results:**

Seven CMO configurations were observed empirically. Findings highlight that a strong coalition of champions, facilitated by cohesion, collaboration, and a shared vision, enhances mobilization. Champions influence QI by prioritizing objectives, reducing perceived effort, and leveraging data to justify change. Their leadership and credibility increase motivation, while their on-site presence helps them anticipate barriers and adapt strategies. Champions initiate small-scale testing to refine interventions and engage peers. Effective communication of results sustains momentum. Ultimately, a champion’s ability to engage the right individuals at the right time, build trust, and provide mentorship determines the success and sustainability of QI initiatives.

**Conclusion:**

This study underscores the complexity of implementing change in primary care organizations. The refined program theory enhances understanding of how champions contribute to successful change implementation and how their behaviours can be adapted to local contexts to optimize outcomes. These findings support a context-sensitive approach to leveraging champions as facilitators of change.

## Background

The health system, particularly primary care, is constantly evolving to adapt to technological innovations and demographic shifts, along with increasing care complexity [1, 2]. As the key entry point for care, primary care is essential for coordinating care and preventing and managing diseases, thereby ensuring overall system efficiency [3]. Investing in primary care—through improved team training and the integration of new technologies—is crucial to maintaining service quality and meeting growing population needs [4]. Quality improvement (QI) is increasingly recognized as one of the best options for adapting the healthcare system to its environment, improving its processes, and effectively responding to evolving patient needs [5]. QI uses iterations to optimize processes and make improvements based on what works in practice. Research highlights the positive impact of QI in enhancing healthcare efficiency and general patient experiences [5–7]. Consequently, integrating QI initiatives may improve the adaptability and proactivity of the healthcare system [5–8].

The QI context highly influences the chances of success of the process [6, 7]. Contextual factors include alignment of the project with organizational priorities, resource availability [8, 9], stakeholder engagement [10], and implementation and integration [11]. Much remains to be done to understand why some QI initiatives succeed in achieving and sustaining change while others do not [12]. Many argue that a deeper understanding of the mechanisms driving QI could help bridge these gaps [13–15]. Part of the answer lies in the relationships between team members that facilitate QI. In Quebec and Ontario, the implementation of QI in clinics is shaped by leadership commitment, openness to change, and a culture of collaboration and continuous learning, although QI is sometimes perceived as additional work in certain settings [16, 17]. Systemic factors—most notably government policies, accreditation requirements, resource availability, and facilitation support—significantly influence uptake. The contrast between provinces illustrates this dynamic: Quebec has benefited from provincial QI agents, while Ontario has struggled with limited integration across physician and non-physician health services [16]. Across both provinces, reliable data infrastructure, professional development opportunities, and incentives play a key role in enabling sustained QI efforts primarily grounded in PDSA cycles [18].

Having a champion who actively contributes to team engagement can help illuminate these contextual factors and could play a major role in the success of QI [19]. The presence of a champion appears to be a critical factor in the successful implementation of change [20]. The champion is an individual who leads or endorses change in their organization [21–24]. Champions support the implementation of change and are generally well-connected within their organizations and communities, which gives them the social influence needed to promote change [23]. Although studies show that a champion is needed to foster change, little is known about how they endorse significant changes and why this is efficient [25].

The specific behaviours of a champion that impact the success of a QI initiative remain poorly understood. The promotion of QI by a champion is a complex intervention that is deeply embedded in the QI process. Nonetheless, the champion’s involvement is often oversimplified in empirical studies [26]. Consequently, it is challenging to isolate the specific impact of a champion from other elements that can influence the overall QI process. In recent years, some studies have attempted to conceptually explore the causal relationship between the presence of a champion and the success of changes [19, 27, 28], but none have conducted empirical testing to improve understanding.

A more comprehensive understanding of the champion’s actions and attitudes as an intervention to promote the QI implementation process in a context could provide clarity on what can be specifically attributed to the champion in the QI process. This study aims to fill this gap by proposing a refined program theory about champions’ behaviours during the QI process in interdisciplinary primary healthcare clinics.

## Method

The study design consisted of a retrospective, multiple-case study as described by Yin [29]. This method allows for the emergence of detailed descriptions of contexts and processes in their natural environment [29–31]. The study uses a realist evaluation (RE) approach to identify “what works, in which circumstances, and for whom” [32, 33]. This design is useful for evaluating complex and constantly-evolving interventions such as the champion’s behaviours [34]. The realist approach aligns with the philosophical current of critical realism [34–36]. RE utilizes Context-Mechanism-Outcome (CMO) configurations as a heuristic tool used to describe the causal relationships between the program (in this case, the champion intervention) and its outcomes in context. The context represents the set of pre-existing elements of one or more aspects of the program [37, 38]. The context has been conceptualized as the relational and dynamic features that shape mechanisms throughout the QI implementation [39]. The outcomes are the observable results of a program following one or more mechanisms activated by the champion’s intervention in a context, and the mechanisms are underlying elements that can be activated or not depending on the context and the program [36, 40–42]. These mechanisms enable a causal link between a context and an outcome in the form of a CMO configuration [36, 43]. Mechanisms are generally underlying and, thus, not visible, as they mainly consist of reasoning elements or reactions observed in a context that result from one or more aspects of the program [36, 38, 44]. CMO configurations establish a conceptual linkage between context, mechanism, and outcome by highlighting the underlying causal relationships that bind these elements together [35, 40]. The program theory integrates all the CMO configurations [38] and is refined through the RE process into a middle-range (or intermediate) theory or a refined program theory that describes how the intervention in context modulates the components to produce a desired transformation [37, 38].

RE typically consists of four distinct steps, applied iteratively and non-linearly. The first step involves selecting an initial program theory. The theory used in this study was derived from a literature review and specifically developed to address our research question [28]. The initial program theory includes 17 CMO configurations and has been published elsewhere [19]. This multiple-case study represents steps 2, 3, and 4 of the RE approach: data collection, data analysis, and validation of the refined program theory. The multiple-case study design is consistent with an RE approach to refining a program theory [29–31]. This structure is appropriate for fostering the emergence of the elements of context, mechanisms, and outcomes used in RE. The retrospective design enables the selection of cases based on the champion’s roles, actions, and outcomes during the QI implementation process. The overall method supports the in-depth exploration of program theory to explain the champion’s impact on QI [29]. The targeted sample size ranged fromfour to six cases, with six to eight participants per case, depending on CMO configuration saturation. Saturation was defined as the point at which the inclusion of additional participants or cases no longer yielded new CMO configurations or contributed to a deeper understanding of the cases or existing configurations [45]. The cases were chosen iteratively based on their informative potential and ability to represent different contexts [46]. This allowed us to compare the differences and similarities, test rival explanations and, thus, increase the theoretical validity of the results, while helping to explain the variations in CMO configurations [47].

### Settings

The cases were defined as a champion involved in a QI process in a primary healthcare clinic. The QI process needed to correspond to the definition proposed by Backhouse for its simplicity but comprehensiveness [48]. According to this definition, QI is a principle-based, systematic, and continuous approach to solving healthcare problems using PDSA (Plan-Do-Study-Act) cycles [48]. Cases were identified from a convenience sample of various QI processes completed or in progress in a primary healthcare clinic between 2016 and 2023. An initial step involved selecting ongoing or past QI initiatives. We then engaged with the teams and change agents, to narrow the selection to cases where a champion had particularly distinguished themselves through their leadership during the QI process. Cases were selected to ensure maximum variation across contexts (e.g., use of external facilitators, top-down versus bottom-up approaches). This strategy was intended to capture the diversity of cases [34]. This theoretical sampling strategy was designed to strengthen the internal validity of the analysis and to foster a deeper understanding of the causal mechanisms underlying the effects of champions. In this study, a champion is an influential and respected individual who actively advocates for change, mobilizes others, and facilitates the adoption of new practices within an organization [23]. According to this definition, we operationalised the concept of a champion as a professional that supports the QI initiative, is actively involved in the various stages of the QI, and is recognized as a champion by at least one of their peers.

### Participants

The inclusion criteria were very flexible and designed to select participants who could best inform and develop CMO configurations for each case. Participants were chosen to represent various perspectives; including both supporters and skeptics of the project, who are expected to respond differently to the champion’s behaviour and from a different professional background. Each case included four to eight participants, including at least one champion and three peers (clinical professionals and administrative staff). In most cases, a change agent and a manager were also included. The number of participants per case was determined based on saturation of CMO configurations. To be eligible, participants had to be active members of a primary healthcare clinic team when the clinic introduced the QI process. All participants signed a consent form that was authorized by the research ethics board of the *CISSS de la Montérégie-Centre* (MP-04–2022-653).

Change agents were defined as a person external to the primary healthcare clinic who facilitated the QI process [23]. Throughout the project, change agents had the opportunity to observe interactions among team members, the roles played by champions, and the outcomes of these interactions. They attended QI meetings and provided support for the project.

Peers were defined as healthcare professionals (physicians, nurses, pharmacists, respiratory therapists), administrative staff, or managers who were involved in the QI process. They were recruited on the basis of theoretical sampling [34, 49] and based on the perceived (by the change agent, champion, and other peers) evolution of their attitudes, intention to change, and behaviours during the process.

### Data collection

Data were collected using documentary research, semi-structured interviews, and a logbook. Data collection comprised four stages carried out in an iterative, non-linear process. Consequently, data analysis was carried out in parallel with data collection and influenced participant selection and the data collection tools used [35, 38, 40].

The document review enabled a basic understanding of how the champion was involved in the QI and the general context of the primary healthcare clinic. All documents related to the QI project were consulted, including those relating to meetings with the clinical team (agendas, presentations, meeting notes, decision-support tools), action follow-up (who did what, when, materials developed, indicators chosen), QI outcomes or indicators, and any other available documents that may have provided some understanding of the QI process within each case. These documents enabled the research team to create an overall portrait of the QI process, provided insight into the context, clarified the collection tools, and enabled triangulation with other data during the analysis. Analysis of the available documents allowed for the generation of the vignette that considers the initial program theory as well as the general context of the case [50]. The vignette was used during the semi-structured interviews to encourage participants to use abduction [50]. Abduction consists of inventive thinking that allows one to imagine the existence of hidden mechanisms used by the participants [51, 52]. The research team applied retroduction by iteratively questioning what other underlying mechanisms could plausibly explain and connect the observed Context and Outcome, and r efining these hypotheses in light of empirical data to construct a robust program theory [53–55]. Retroduction is a key concept in RE that combines inductive and deductive reasoning to produce an explanation for observed patterns to produce a theory [53–55].

Individual semi-structured interviews were conducted first with the change agent, when there was a change agent implicated. In the case where there was no change agent, an informal interview was conducted with the champion before the first interview to allow contextualization of the vignette. Then individual semi-structured interviews were conducted with the champion and finally with peers and managers. Change agents and champions have a good overview of and are able to understand the QI process, the context, the champion’s behaviours, and the outcomes and can identify pivotal moments in the QI process. Interview guides were developed to encourage the emergence of rich, detailed descriptions and were revised to ensure the most relevant elements to refine the program theory were captured [50]. The interviews lasted a maximum of 90 minutes. This approach aimed to leverage reasoning skills to uncover the hidden underlying mechanisms connecting the contextual elements, the champion’s behaviours, and the outcomes [50].

Champions and peers were interviewed individually using the same structure and step as the change agents. These interviews allowed us to deepen our understanding of the champion’s behaviours, motivations, and vision of the QI process and explore perceptions of the impact of the champion’s behaviours on each of their peers. After each interview, the vignette and interview guide were refined to reflect alterations made in the program theory [50]. Interviews were conducted in a specific order—first with change agents, then with the champion, and finally with peers—following an iterative logic. This order allowed for the progressive refinement of the interview guides, ensuring that peer interviews were oriented toward the exploration of underlying mechanisms and the refinement of CMO configurations.

### Data analysis

Data analysis was conducted iteratively alongside data collection, enabling refinement of the vignette, data collection tools, and analysis methods throughout the process. Interviews were recorded, transcribed, and validated. Subsequently, the data were coded with NVivo using a direct content approach to identify contextual elements, mechanisms, and outcomes [56, 57]. Parallel coding was carried out [58, 59] to reduce subjectivity and ensure credibility. The coding grid was developed from the initial program theory and revised iteratively [32]. Emerging themes were organized in the form of Context, Mechanism, or Outcome, and then regrouped as CMO configurations for each case [60–62].

Cases were first analyzed separately (intracase), followed by cross-sectional (intercase) analyses [29, 49, 63]. Cross-case analyses identified similarities and differences between cases. The configurations observed in each case were then compared to understand common and specific elements. This step was built and validated by the team to elicit the CMO configurations specific to each champion as well as those that were common across cases and contributed to refinement of the initial program theory [35, 38, 40, 64]. Retroductive analysis then guided the explanatory process, allowing us to move beyond descriptive patterns toward the refinement of CMO configurations.

### Validation of the refined program theory

The refined program theory including all CMO configurations was validated using focus groups [65]. Participants were recruited based on their knowledge and experience in the dynamics of primary healthcare teams and QI processes. Discussions were conducted using comics to illustrate the CMO configurations, followed by a question period where participants were invited to comment on, question, complete, and discuss the configurations [65]. This approach facilitated open dialogue among participants, enabling us to focus on CMO configurations without explaining all RE principles to participants. It also simplified explanations for participants, fostering an effective member-checking process. These discussions were used to validate our interpretations and refine some of our conclusions. In total, three focus groups were conducted with a total of 12 participants drawn from the cases studied and from diverse Canadian healthcare and research settings, representing different areas of expertise such as QI, primary healthcare and knowledge transfer. The involvement of experts and experienced practitioners facilitated reflection on contextual nuances and on the potential limitations of our findings related to case selection.

## Results

Overall, data were collected from four cases with a total of 20 participants, including change agents, managers, healthcare professionals (including champions), and administrative staff. Some participants contributed to two cases (Cases 1 and 2), because they are involved in the QI project of two different cases from the same region. One individual interview per participant was conducted between March 2022 and December 2023. The characteristics of each case are summarized in Table 1. In most cases, the role of the champion was informal, emerging organically, though occasionally recognized by peers or supervisors. The cases were from primary healthcare clinics in Quebec (3) and Ontario (1). The *aim* refers to what each team sought to achieve through their project. *Data utilization* varied across cases; in most cases (3) data were used to establish general project directions and monitor general progress (less than 4 times a year and one team reported using data continuously to inform real-time adaptations throughout the project. The number of participants interviewed per case is also provided in Table 1. Seven CMOs were constructed to elucidate how a champion in a primary healthcare clinic can foster the QI process.

**Table 1:** Cases description

	Case 1	Case 2	Case 3	Case 4
Champion’s profession	Respiratory therapist /Pharmacist	Respiratory therapist	Physician	Physician
Aim	Implement a care trajectory for chronic obstructive pulmonary disease	Implement a care trajectory for chronic obstructive pulmonary disease	Implement the advanced access model	Enhance cardiovascular prevention for diabetic patients
Multisite	No	Yes	No	No
Rural/Urban	Rural	Rural	Urban	Urban
Province	Quebec	Quebec	Quebec	Ontario
Implication of a change agent	Yes	Yes	Yes	No
Data utilisation	General project directions	General project directions	Inform real-time	General project directions
Participants (n)	8	8	6	4
Years	2017–2018	2017–2018	2019–2022	2023-in progress

### Configuration 1: effective communication of the QI objective

Characteristics of the environment, such as cohesion, collaboration, and a shared vision of the mission (C), foster team mobilization towards a common goal (M) and enable the establishment of a coalition of champions (O). The champion can further potentiate change by engaging the right people and creating a coalition of champions. A coalition of champions is an alliance between several team members who work together towards a common goal, in this case, the QI process: *A champion who solicits the right people promotes culture change. That’s the basis of choosing the right people, a more natural way of guiding or having a vision of what’s to come; a broader vision allows for a culture change that may involve not just one theme but also a culture change that will be broader.* (Nurse, Peer, Case 1)

Many participants stated that an alliance of individuals from varied backgrounds—spanning different generations, educational levels, and values—enables the creation of a robust coalition of champions. The coalition effectively fosters change across the groups that members belong to: “We’ve brought in a new physician with us for a month. I made the request. I wanted a younger physician to help with the influence of younger physicians at the clinic. So I went and got myself a peer who had a similar vision of access to mine.” (Physician, Peer, Case 3)

Changes that align with the well-being of patients appeared to be one of the means that can be promoted to facilitate adherence to the vision: *We do it for the patients. Any conflict management, we will try to refocus around the user’s well-being. So, by having them around the table, and who contributes, who adheres to the vision to which they contribute. Of course. I think it’s very facilitating for a champion.* (Manager, Case 2)

All participants defined the champion as a trusted peer able to obtain the support of managers. “Having a positive influence on the team without being seen as a boss, a manager, or part of the administration is essential. However, this depends on the level of autonomy they are given in decision-making processes. If the manager supports the champion, things will go smoothly.” (Focus group 2). Having a competent peer, the champion, who understands the reality of the field as well as the challenges and barriers to change helps to build trust within the team and facilitates communication of the benefits of the QI process. The leadership skills of the champion as well as their knowledge of the reality of their peers (e.g., being in the same profession) help them gain the trust of their peers. Peers felt that champions have valuable inner leadership traits needed for changes, which increases mobilization towards the objective: “So the foundation of any project must be a relationship of trust. We were talking about credibility too, but it’s about saying that we want to work together towards the same goal, and if we don’t trust each other, we fail miserably.” (Manager, Case 1)

One champion explained that, initially, she had to gain the trust of her peers regarding her clinical skills to bring them on board with the change: “I had more of an impression of needing to prove myself to establish a bond of trust… they needed to understand what the added value was for them, without adding an extra workload.” (Respiratory therapist, Champion, Case 1)

### Configuration 2: QI objective

All the QI processes started with an improvement objective. An improvement aim is a broad, aspirational statement describing the overall desired impact of a change effort. An improvement objective breaks that aim into specific, actionable, and measurable short-term targets. The champion communicates the objective of the QI process (C) to increase the perception that it is a priority and has a low effort/high impact ratio (M). This increases motivation to work towards the objective (O). The champion, as a dedicated individual, passionately believes in the objective and can inspire others to join the cause. Many peers spoke about the champion’s enthusiasm being contagious. The champion’s influence is magnified by their credibility and communication skills with peers and managers, enhancing the perception that the issue is a high priority and will have significant impact.

A change agent described the champion and their effect on the team: *These are passionate people, and they do it for the patient. So, they put themselves at the service of a cause that is greater than themselves to advance the project, ultimately to improve, in this case, the trajectory in COPD and patient care services. The fact that they are true leaders, you can feel that they are not doing it for themselves, they are doing it for the cause out of conviction. And that, people can feel it. And when they feel that, both clinicians and managers, it awakens in them the same passion, it motivates them.* (Change agent, Cases 1 & 2)

When the objective of the QI process is a priority for the organization (C), it can increase individual prioritization (M); but when there are barriers or when the effort seems too high, the objective is not enough to mobilize the team. It takes a local team member to bring the project to life. Some cases had very strong organizational support to achieve the QI process. These managers were appointed as project facilitators, but they said that without strong local leaders in the field, i.e., the champion, the project would not have been the success it was: *But I think if the medical staff wasn’t ready, and they didn’t want to get involved, it would probably have fallen flat. [The manager] wouldn’t have been able to do it alone. You know, we had to make sure that, with responsible physicians who engage with them beyond just involvement, the necessary resources were in place.* (Change agent, Cases 1 & 2)

Moreover, use of data by the champion to show why the objective (C) should be prioritized helps increase its credibility (M) and helps promote prioritization (M) of the objective and mobilization of the team (O). “He said, ‘Yesterday, to tell you the truth, we had 22 hooks, 22 patients who called in for an emergency that couldn’t be seen.’ So, it’s clear that the people on the team understood that this was really a critical situation.” (Physician, Champion, Case 3)

### Configuration 3: diagnoses of the system

The presence of a champion who identifies barriers and can engage the right people and obtain the resources to reduce these barriers (C) helps decrease the perception of effort required for change among peers (M), which reduces resistance to change (O). The champion, as a representative in the field who understands the setting and can identify potential barriers to the proposed changes, significantly enhances the change management process. Being physically present in the environment enables the champion to anticipate and address obstacles effectively. This hands-on approach facilitates a more immediate and informed response to challenges, thereby streamlining the path to successful change implementation. A few participants named having a champion of the same profession as being preferable, as they share similar references and vocabulary: *Anticipating and solving barriers, I think that can increase the receptiveness of clinicians. Because if we take the time already to think about what the apprehensions of certain people are, things that we already identify that could harm the project, that is already stated, that we have thought about this or that, that we have solved this or that problem, well clearly, that can make the clinicians who are involved in the project want to mobilize because there is a portion of things that we had to anticipate that have already been thought about or mentioned, at least, which means that it can perhaps facilitate the achievement of all that. And I would have linked it to reassurance too.* (Pharmacist, Peer, Case 2)

In two of the cases, the change needed approval from a manager to be tested and to free up the resource. Then, the ability of the champion to clearly communicate the need to the manager facilitated its approval and thereby promoted the implementation of the change. The manager’s openness to the change also greatly facilitated the overall QI process: “He made sure that his resources are freed up, and it’s a gymnastic effort to free up resources.” (Manager, Case 1)

The perception of the effort required to change was identified as an important factor influencing the intention to change: “But I think it also depends on the ask for the person. If it involves a lot of the clinicians’ time to do it, it will be challenging.” (Physician, Peer, Case 4). A champion explicitly states their intention to reduce the effort required for the change to encourage the involvement of their peers: “We are always able to manage to find strategies for those who don’t want to take on more.” (Physician, Champion, Case 3)

### Configuration 4: navigating towards improvement: pragmatic choices

The presence of a champion in the environment allows for testing the change on a small scale (C), which allows them to better understand the change (M) and to adapt the change to the needs (M) so there is less resistance to the change (O). Most of the champions were the first to test the change in their setting or were implicated in testing the change. Their presence allowed for small changes, testing, measuring, and adapting the change idea on a small scale before proposing the change to their peers with a clearer understanding of the expected results in their environment: *She was often the first to try out new things, and she was really, really good at it. When we wanted to convince people, we would say, ‘You know, we’re going to work with physicians, and it’s working well, and…’ She was able to precisely share her experience, not just the positive aspects necessarily. She had, you know, a lot of openness to say, ‘This works. That doesn’t work. This…’ So, she was truly a champion in that regard.* (Change agent, Case 3)

The champion, through their own experiences and by listening to their teammates, can help identify possible issues and adapt the change when required. This can facilitate the QI process because, for some people, experimenting with issues could create reluctance towards change: *Actually, we’ve learned in our computerization processes that we need to go low, go slow. So, crashing the whole organization is not good. Because when you’re working with physicians, you have one chance to make it work, not two. So often what we do is we test our processes on a very small scale with a physician who is tolerant to bugs. Because doing computer processes necessarily involves bugs.*(Physician, Manager, Case 1)

Moreover, the fact that champions are present on-site allows them to hear and see the barriers encountered by their peers. This proximity allows for exchanges that enrich the proposed change in the context, thereby increasing the perception of the change’s credibility and increasing the motivation to change: *Feedback, based on my personal experiences, has facilitated consensus. And let me explain a bit because it might not be the most obvious connection between the two, but by providing feedback, it allows for a further discussion, a more detailed discussion of each person’s vision, and the successes and failures in order to achieve something more interesting.* (Pharmacist, Peer, Case 2)

By testing the change in their environment, the champion demonstrates that the change is well adapted to the context (C), which increases the perception that the change is positive and credible for peers (M) and encourages motivation for change (O): “I’ve shown that it worked, that I was seeing more patients, that’s all there is to it, and for sure, to change that, people need to see the added value and have the willingness to do it.” (Physician, Champion, Case 3)

### Configuration 5: team in action

When one or more other peers are ready to change, the champion can include them and modulate the pace and the amplitude of the change (C) to reduce resistance to change (O). The champion who is highly sensitive to the needs of their peers is able to include the right peers at the right moment and to propose a change adapted (C) to reducing fears and the perception of effort (M) for that peer.

One change agent spoke about situations that contrasted with the attitude of a successful champion: *So, there was this physician who was truly a champion but not at all a leader. She wasn’t able to get everyone on board because she was very focused on performance. If you don’t do it exactly like this, then it’s not right. So, it put a lot of pressure on people who may have wanted to get on board, but at the end, they struggled more.*(Change agent, Case 3)

Similarly, one champion mentioned the added value of taking time to build the trust of certain peers and slowly bring them into the collaboration: “So, I go more slowly. And I ask their opinion more before changing anything. I do it less myself, it’s slowly. When it’s their patient, I have to go more gradually, I adapt myself a bit.” (Pharmacist, Champion, Case 1)

At the same time, many participants named the importance of communication from the champion to feel that the project is progressing. Most stated that positive outcomes are the best motivation to change, because ultimately, individuals are motivated by the promise of the change’s results. The communication of gains by the champion to all team members (C) demonstrates the impact of the change and maintains momentum (M), which helps maintain the motivation to change (O): “Project management that addresses technical problems as they arise. But it’s really motivating because ultimately, there are results for the patients, and that speaks to clinicians and managers, who all generally have that drive in the healthcare field.” (Change agent, Case 1)

In the same way, the champion can motivate their peers by providing positive feedback about their participation (C), which is rewarding (M), maintains motivation for the change, and helps sustain the process (O): “When you receive positive feedback from your peers that values what you do, it gives you the fuel to keep going.” (Pharmacist, Champion, Case 1)

When the team starts to get involve, the more the change is perceived as complex to adopt, the more support, mentorship, or training from a champion are helpful (C) to reduce fears and the perception of the effort required (M), which reduces the resistance to change (O). In one case, the champion offered mentorship to a younger physician who felt insecure during walk-in clinics by acting as a safety net to encourage them to try to change and to demonstrate their ability to do it by themselves.

### Configuration 6: integration to simplify behaviour change

Even when the context of a change is favourable and individuals desire to change, behaviour change remains difficult. Thus, the use of reminders throughout the change (C) to reduce the effort required for the behaviour change (M) reduces the resistance to change (O).

Participants in all cases emphasized the benefit of perceiving a low effort/high impact ratio. In general, the presence of the champion helped to contextualize the change and reduce barriers. In one case, the major champion strategy was based on reminders to ease the change: “You know like it’s we get a message directly in the patient’s chart to say, hey, this person has not been on statins. When you get a direct reminder like that, it helps.” (Physician, Peer, Case 4) In this setting, most of the physicians accepted the benefits of the change because it was a simple evidence-based change. The difficulty was integrating this change into their practice. A manager in another case also spoke about how difficult it can be to change a behaviour: “Changes initially take us out of our comfort zone and may require more work to adapt. But I think the important thing is that the patient receives the best care. And also, after a while, we get used to the new procedure, which becomes easier too.” (Manager, Case 1) Sometimes, just by their presence and enthusiasm, the champion can help others remember what they should do and help to make the change: *If you have a champion, if you’re someone who is talking about it all the time, bringing it up all the time, reminding you all the time. That helps. Sure, that helps sometimes. It’s just there are so many things going on you just don’t remember. There’s so many priorities*. (Physician, Peer, Case 4)

### Configuration 7: normalization ensures sustained change

At the moment of data collection, one of the four change initiatives was still in progress, two had been sustained, and although one project had concluded, there was still some interest in maintaining its outcomes. To increase the sustainability of the positive results of the QI process (O) the champion can put elements in place that will allow the change to last over time (C) following normalization of the change (M). This is because sustainability is a significant challenge, as mentioned by many participants: “Even if it’s a good project, unless you have people devoted to doing it, it’s really difficult to sustain. And I think that is something that we recognize at our unit, that sustainability, even if it’s a wonderful project, it’s hard.” (Physician, Case 4)

Standardization can take time, which means that personnel turnover must be planned for. In one case, one of the champions left and was replaced by another person who did not have the same level of knowledge. However, this environment included a coalition of champions ready to support the new employee in their learning and allow them to continue the processes in place. In this case, staff turnover required adjustments and delayed new processes, but it can also have positive outcomes when it involves highly resistant individuals leaving the environment. This means that champions should view the time required for standardization as an opportunity to support change by being patient and implementing contingency plans and training for new staff.

The champion can also help in this phase by recruiting the right people or by asking the manager to incorporate the change into processes: “It is necessary that within the [clinic], they embed it significantly. The ones where it works the best and is sustainable, it’s at the level of high governance, it has to be the head of the [clinic] or the person assigned to that approach who stays.” (Champion, Case 1)

During the discussion groups, participants emphasized the importance of normalization, where innovative practices become the standard within the organization. But some added that this does not depend solely on the champions but rather on adoption by all team members.

*I don’t think it’s solely up to the champion to ensure normalization. Normalization also comes with generalization across the entire structure. Sure, maybe the champion can uphold a sort of moral contract, keep an eye on things to make sure everyone stays aligned.* (Focus group 1)

They also pointed out that it is not necessarily the champion’s responsibility to plan their own exit strategy, but rather for the entire group to take ownership of and sustain the implemented improvements.

## Discussion

This RE revealed key behaviours that a champion can take to improve the chances of success for QI processes. The presence of a champion by itself can be seen as a positive indicator of the relevance of the QI in the organization. The motivation for change varies from one individual to another and depends on several factors [66]. To increase individuals’ motivation to get involved in a change, it is essential to promote a positive perception of the effort needed and potential impact, to propose a credible path to success, to adapt to the context and prioritize, to reduce fears about change, to maintain momentum, and to facilitate integration of the change. Standardization of specific processes also helps ensure the sustainability of the change. The implication of the champion for the standardisation was not as clear as for other steps during this study, which aligns with recent findings showing that sustained improvement is more likely when implementation strategies are well-embedded in routines, supported by leadership, not necessarily a champion, and aligned with organizational capacity, systems, and resources [67]. However, the champion’s presence alone is not enough to enable the adoption of the change by the entire team or to maintain changes over time. Nevertheless, the champion’s presence creates favourable conditions for the QI process.

Compared to the initial program theory [19], the refined program theory contains fewer configurations (seven compared to seventeen), but they are significantly more complex and incorporate more elements. Several champion behaviours were grouped together because they were based on very similar mechanisms. For example, in the initial theory, having a champion from the same profession as their peers was expected to enhance credibility and strengthen peers’ confidence in their own ability to make the change. While our findings supported the importance of peers’ confidence in their ability to change, the link between professional similarity and the champion’s credibility was not confirmed. In fact, only one participant mentioned that sharing the same profession might increase the champion’s credibility. The peers acknowledged the importance of credibility, but without emphasizing professional background, and none suggested it would make them more likely to adopt the change. The factors contributing to a champion’s credibility appeared to vary across individuals. This approach also allowed us to better reflect on what we observed in reality by highlighting the importance of the group and the need to form alliances with other actors. The mechanisms we observed—reducing fear, sustaining momentum, normalizing new practices—are consistent with work linking inclusive leadership, psychological safety, and engagement in QI [68, 69]. Overall, the refined program theory offers a more systemic and cross-cutting perspective, nuanced by the role of context and emphasizing the importance of rallying peers rather than focusing solely on an individual. 

Typically, in QI, champions inspire, change agents orchestrate, facilitators provide support, and coaches develop skills, together forming a synergistic ecosystem that promotes sustainable and effective change [70, 71]. Our findings align with this framework but suggest more fluid boundaries between these roles. In practice, the success of change depends primarily on addressing the needs of those targeted by the initiative, rather than on the formal title or role of the individual driving the change.

The importance of a champion, integrated into the environment and able to ease the QI process in the field, is undeniable in these data. The findings revealed that some of the champion’s interventions described in the CMO configurations can also be made by actors other than the champion. Interventions like prioritizing the project, communicating the objective and progress, providing data, and normalizing the change can easily be done by a skillful manager or a change agent. However, some actions must be done by someone in the field who has close relationships with their peers and good comprehension of their reality. An important behaviour that the champion makes, and that must be made by a champion, are to test the initial version of the change and to give insights into the organization and the context of the change. In a QI process, someone must be willing to do the initial testing and be open to the change, usually a champion alone or with peers. This mirrors recent evidence showing that effective champions engage in participatory leadership, involving colleagues in iterative testing and real-time adaptation of innovations, thereby fostering a shared sense of ownership and surfacing contextual nuances (e.g., through engaged feedback loops) [72]. The champion will have to adapt the change to the setting and adapt the overall QI process to the cultural environment. The champion also understands which of their peers can test the change with him or after some iteration of the change and which are not ready for that. Therefore, to be efficient, the champion must understand the overall cultural environment and be sensitive to individuals and the team. In alignment with this, Demes et al. (2020) identified critical champion attributes—which include influence, ownership, physical proximity to change points, persuasiveness, perseverance, and participative leadership—that empower champions to navigate institutional silos and leverage professional networks to continuously tailor implementation to local workflows and social realities [24] This ability is likely the most valuable factor, because it helps reduce the chances of failure, discomfort, or fear surrounding the change. Lowering people’s fears and respecting their need for control and security are all elements that enable champions to reduce resistance to change and create a feeling of security, as previously reported by Nembhard and Edmondson, who explored the link between being implicated in a QI process and psychological safety [73].

The champion also helps with the alignment of priorities and vision between those in the field and the manager. Fluidity of communication between the champion and the manager is a key contextual factor for the success of the QI process [74, 75]. The champion’s ability to secure the support of resources from the manager is essential to facilitate the entire QI process. This aligns with the well-established evidence base highlighting the importance of the champion collaborating with managers and effectively obtaining the necessary resources [74, 75].

This study represents the first RE focused on exploring the mechanisms through which champions influence QI processes. The results provide insights into primary healthcare clinic teams’ reactions to champions’ initiatives and prioritize the in-depth analysis of causal mechanisms in the context of QI in primary healthcare clinics. Consequently, our results provide an in-depth understanding of the champion’s role as it unfolds in this particular context, while acknowledging that this understanding may not be transferable to other settings or contexts. However, they contribute to a refined program theory, which describes the champion’s actions and outcomes and the causal relationship between them. This theory could be tested and refined in other settings in future studies to deepen our understanding of team dynamics. The refined program theory explains mechanisms that lead to motivation for the QI process. It suggests that the champion’s ability to adapt to their peers’ individual contexts helps reduce their resistance to change or increase their motivation. Thus, knowledge of their peers and understanding of their vision and reality is necessary for the champion’s effectiveness. Our results suggest that, although the champion is needed, they are not sufficient on their own for the implementation of the project. Indeed, a champion minimally needs the support of managers, feedback, time, and communication skills. The evidence from this assessment is in alignment with other studies [74, 75].

This study is the first empirical investigation of the causal relationship between the champion’s presence and the implementation of QI processes. It elucidates the mechanisms that lead to greater efficiency of projects with a champion as well as highlighting limitations. The strengths of our study lie in the RE approach in general, which enabled us to consider many of the complexities of the problem and allowed for an in-depth study of this causal relationship. Also, to ensure the trustworthiness of the outcome, the RAMESES guidelines were used, and several approaches were taken [76–79]. Data were collected from various sources (documents, interviews, and focus groups) and various perspectives (champions, change agents, and peers), allowing for triangulation of methods and data sources [80]. The detailed descriptions of context and the data analysis process allowed for transferability and dependability. To improve the theoretical validity of the results, alternative explanations were systematically explored. Theoretical saturation was achieved despite the relatively small sample size, which enhances the validity and reliability of the results [81, 82]. The research team contributed iteratively to the development of the interview grid and the analysis as well as the research diary to help the first author consider her own bias. The use of focus groups also allowed for validation of interpretations and helped to add nuance.

However, this study had some limitations. The retrospective design is susceptible to memory and amplification biases [83] as well as potential social desirability bias, which may have compromised the credibility of the findings [84, 85]. To mitigate these biases, various measures were implemented, including using a vignette for reference, triangulation of data sources, and examination of alternative explanations during the analysis process. Third, general knowledge of the QI process and how it should be integrated in primary healthcare clinics is growing rapidly in Quebec and Canada; therefore, the role of the champion could change in the future. Finally, the proposed theory is only valid for explaining the causal relationship of the champion’s behaviours in the context of the QI process in primary healthcare clinics in Canada. Further research in other settings would be beneficial in assessing the applicability of this theory in different contexts.

## Conclusion

This study clarifies the intricate causal relationships and underlying mechanisms through which champions mitigate resistance and bolster motivation for QI processes. It establishes a strong foundation for further exploration into the pivotal role of champions in fostering a culture of QI within interdisciplinary primary care clinics. Additionally, this research contributes to the existing body of knowledge by providing valuable insights into the distinctive characteristics that define successful champions in the domain of quality enhancement efforts.

## Data Availability

The datasets used and analyzed during the current study are available from the corresponding author on request.
